# Prevalence and diversity of haemosporidian parasites in the yellow‐rumped warbler hybrid zone

**DOI:** 10.1002/ece3.4469

**Published:** 2018-09-12

**Authors:** Camille‐Sophie Cozzarolo, Tania Jenkins, David P. L. Toews, Alan Brelsford, Philippe Christe

**Affiliations:** ^1^ Department of Ecology and Evolution University of Lausanne Lausanne Switzerland; ^2^ Fuller Evolutionary Biology Program Cornell Lab of Ornithology Cornell University Ithaca New York; ^3^ Department of Evolution, Ecology and Organismal Biology University of California, Riverside Riverside California

**Keywords:** avian malaria, elevation, hybrid zone, postzygotic reproductive barrier, *Setophaga coronata auduboni*, *Setophaga coronata coronata*

## Abstract

Parasites can play a role in speciation, by exerting different selection pressures on different host lineages, leading to reproductive barriers in regions of possible interbreeding. Hybrid zones therefore offer an ideal system to study the effect of parasites on speciation. Here, we study a hybrid zone in the foothills of the Rocky Mountains where two yellow‐rumped warbler subspecies, *Setophaga coronata coronata* and *S. c. auduboni*, interbreed. There is partial reproductive isolation between them, but no evidence of strong assortative mating within the hybrid zone, suggesting the existence of a postzygotic selection against hybrids. Here, we test whether haemosporidian parasites might play a role in selecting against hybrids between *S. c. coronata* and *S. c. auduboni*. We screened birds from five transects across the hybrid zone for three phylogenetic groupings of avian haemosporidians *Plasmodium*,* Haemoproteus* and *Leucocytozoon* parasites and quantified intensity of infection. Contrary to our prediction, hybrids did not have higher haemosporidian parasite prevalence. Variation in *Haemoproteus* prevalence was best explained by an interaction between a birds’ hybrid index and elevation, while the probability of infection with *Leucocytozoon* parasites was only influenced by elevation. We also found no significant difference in the diversity of haemosporidian lineages between the warbler subspecies and their hybrids. Finally, intensity of infection by *Haemoproteus* increased significantly with elevation, but was not significantly linked to birds’ hybrid index. In conclusion, our data suggest that haemosporidian parasites do not seem to play a major role in selecting against hybrids in this system.

## 1 INTRODUCTION

Parasite‐mediated divergent selection can be a strong and widespread mechanism of ecological speciation (Summers et al., 2003). It is powerful, as some parasites impose large fitness costs on their hosts, and widespread, as virtually all animals are hosts to a high diversity of parasites (Price, 1980). Variation in infection among populations of the same host species might lead to the evolution of divergent resistance gene combinations and to parasite‐mediated divergent selection (Karvonen & Seehausen, 2012). In areas where divergent host lineages hybridize, recombination across generations of hybrids might break up assemblages of coadapted genes, reducing hybrid fitness relative to parental genotypes (Rundle & Whitlock, 2001). In cases where heterogeneous infections are not the primary cause of divergence, the same mechanism might also reinforce the reproductive isolation by imposing strong selection against hybrids. In addition, if there is strong specificity between parasites and their host genotype, the latter may have evolved specific resistance. Hybrids could thus be vulnerable to parasites of both parental taxa, yet lack the resistance that parental species have coevolved with their specific parasites (Wolinska, Keller, Manca, & Spaak, 2007). On the other hand, in some systems, hybrids benefit from heterosis, as heterozygous individuals have access to a wider range of resistance alleles (MacDougall‐Shackleton, Derryberry, Foufopoulos, Dobson, & Hahn, 2005; Niskanen et al., 2014).

Several cases of parasite‐mediated selection against hybrids have been documented in the literature (Moulia, 1999). For example, crosses of mallards and black ducks are more frequently infected by *Sarcocystis* parasites (Mason & Clark, 1990). In the house mice, *Mus musculus musculus* and *M.m. domesticus* (Derothe, Le Brun, Loubes, Perriat‐Sanguinet, & Moulia, 2001), hybrids are parasitized more by nematodes and cestodes than the parental subspecies (Moulia et al., 1991; Sage, Heyneman, Lim, & Wilson, 1986). Wolinska, Keller, Bittner, Lass, and Spaak (2004) found that the protozoan gut parasite *Caullerya mesnili* decreases the fitness *of Daphnia galeata *×* hyalin*a hybrids, which are significantly more infected than the parental species.

Haemosporidian parasites, and in particular avian malaria parasites, are an important model for the study of the evolution of host–parasites relationships. Their high diversity (at least 900 lineages, Bensch, Hellgren, & Pérez‐Tris, 2009) and their variability in virulence make them good candidates to investigate parasite‐mediated divergent selection. Haemosporidian parasites are also very common in many bird species (Ayadi et al., 2017; van Rooyen, Lalubin, Glaizot, & Christe, 2013; Swanson, Lyons, & Bouzat, 2014) and can reach high prevalence, for example in great tits (*Parus major*) where prevalence was as high as 91% in some Swiss populations (Glaizot et al., 2012). These parasites are represented by the genera *Plasmodium*,* Haemoproteus*, and *Leucocytozoon* and are transmitted by dipteran vectors (Valkiūnas, 2005). There is evidence that avian malaria has a significant effect on the fitness of infected birds by increasing mortality (Atkinson, Dusek, Woods, & Iko, 2000; Marzal, Bensch, Reviriego, Balbontin, & De Lope, 2008; Sol, Jovani, & Torres, 2003) and decreasing reproductive success (Knowles, Palinauskas, & Sheldon, 2010; MacDougall‐Shackleton et al., 2005; Marzal, de Lope, Navarro, & Moller, 2005; Merino, Moreno, Jose Sanz, & Arriero, 2000). However, counter‐examples exist where no strong effect of avian malaria on fitness has been found (Kilpatrick et al., 2006), suggesting that the effects may be dependent on the lineages and host combinations considered. By reducing the fitness of hybrids more severely than the fitness of parental species, haemosporidian parasites have the potential to drive speciation in their hosts, as it has been suggested in macaques (Wheatley, 1980).

To investigate parasite‐mediated speciation, tension zones, a particular type of hybrid zone, are relevant. Tension zones are characterized by a relatively narrow width and stabilized by a balance between selection against the hybrids within the zone and dispersal of parental types into the zone (Barton & Hewitt, 1985). The sources of selection against hybrids in most tension zones are unknown, although the role of parasites in this context has not been extensively studied (Alexandrino et al., 2005; Singhal & Moritz, 2012).

**Figure 1:**
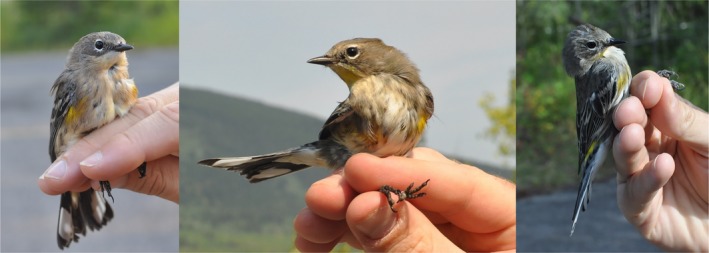
Migrating yellow‐rumped warblers (photograph credit: David P. L. Toews)

One well‐characterized hybrid zone where hybrid fitness is unclear is between myrtle warblers (*Setophaga coronata coronata*) and Audubon's warblers (*S. c. auduboni*) (Figure 1). These two subspecies interbreed in a narrow region across the Canadian Rocky Mountains (Hubbard, 1969). Brelsford and Irwin (2009) tested birds in the hybrid zone for evidence of assortative mating, and found a pairing pattern consistent with random or very weak assortative mating. Brelsford and Irwin (2009) also found that selection against hybrids is necessary to maintain the observed linkage disequilibrium and cline width. The mechanisms of this inferred selection are still unclear. Here, we test the hypothesis that haemosporidian parasites may play a role in the selection against *S. coronata* × *auduboni* hybrids.

Patterns of prevalence and diversity of haemosporidian parasites can vary according to several different biotic and abiotic factors, yet the underlying factors are still poorly understood. Scordato and Kardish (2014) showed that host species is one of the main predictors of prevalence and diversity, and a better predictor than geography. However, environmental factors also seem to be important, particularly as they will affect vector ecology thereby generating spatial structuring of haemosporidian occurrence (Cumming et al., 2013; Ferraguti et al., 2018; Loiseau et al., 2012; Mendes, Piersma, Lecoq, Spaans, & Ricklefs, 2005; Wood et al., 2007). In blue tits (*Cyanistes caeruleus*), for example, in addition to be specific to each parasite lineage, malaria prevalence seems to vary between host populations, suggesting that either coevolutionary history or environmental variables influence prevalence (Szöllősi et al., 2011). Elevation can also influence haemosporidian prevalence, because environmental variables such as temperature or plant communities change with elevation (Imura et al., 2012; Latta & Ricklefs, 2010; van Rooyen et al., 2013; Zamora‐Vilchis, Williams, & Johnson, 2012).

Here, we investigated the potential role of haemosporidian parasites in selecting against *S. c. coronata* × *auduboni* hybrids. The data set we used is extensive in sample size and geographic area, composed of five independent transects, which allowed us to test hypotheses regarding the geographical structuring of haemosporidian distribution. By amplifying and sequencing a fragment of haemosporidian *cytochrome b* in blood samples from warblers in the hybrid zone, we assessed the infection status and, when possible, identified the parasite lineages present. Using a quantitative PCR protocol, we measured the intensity of infection (parasitaemia) by the most common lineage in the hybrid zone. By these means, we determined whether hybrids had higher haemosporidian prevalence and parasitaemia than pure *S. c. coronata* and *S. c. auduboni*, which could be expected if hybrids were less adapted to resist haemosporidian infection. As hybrids may inherit specialist parasites from both parental subspecies, we also tested for higher haemosporidian lineage diversity in hybrids. However, differences in infections may be affected by environmental parameters as well as bird ancestry, so we also assessed how geographical variables, specifically elevation, influenced haemosporidian prevalence in myrtle and Audubon's warblers.

## 2 MATERIALS AND METHODS

### 2.1 Field sampling

**Figure 2:**
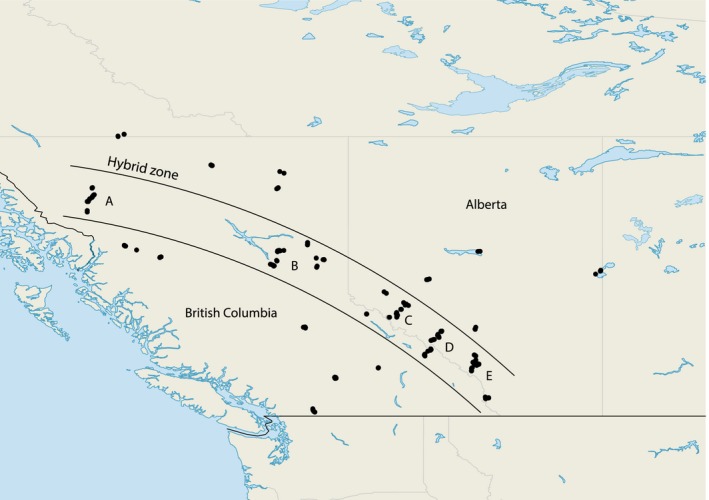
Map of sampling sites (black dots). Letters indicate the five transects

The majority of samples (*n *=* *196 *S. c. coronata*,* n *=* *193 *S. c. auduboni* and *n *=* *228 hybrids) used for this study were initially collected by Brelsford and Irwin (2009). Briefly, we captured warblers defending their territories on their breeding grounds (that is, mostly males), along five transects across the hybrid zone and additional allopatric sites, in Alberta and British Columbia (Figure 2). We took several morphometric measurements and scored five plumage color traits that differ between *S. c. coronata* and *S. c. auduboni*, following Hubbard's (1969) hybrid index: 0 for a *coronata*‐like trait, 2 for an *auduboni*‐like trait, and 1 for intermediate states. The hybrid index is the mean of the scores of the different traits, listed in Supporting Information Table S1. We excluded the sixth trait used by Hubbard (1969), tail pattern, from analysis due to concerns over its repeatability. We also excluded auricular color from analysis in females, because in both subspecies female auricular patches were brown rather than black or gray.

In addition, we also sampled yellow‐rumped warblers during autumn migration in 2015 (*n* = 131), between August 28th and September 12th near Barrier Lake (51.023591, −115.060657, elevation: ca. 1,400 m) in the Kananaskis region of Alberta, Canada. The aim of this new sampling was to catch hatch‐year birds starting their first migration to compare their parasite lineages composition to that of birds that have already been on wintering ground. This site is close to the geographic center of the hybrid zone, but birds captured during migration may have come from distant breeding locations. We captured migrating yellow‐rumped warblers using mist nets with song and call playback, and determined the age of the birds (hatch‐year/after hatch‐year) by examining skull ossification (Norris, 1961). We also took morphometric measurements. The use of the full hybrid index (Hubbard, 1969) is more difficult when the birds do not display their breeding plumage, so in this case, we used a genomic hybrid index (see below). Blood samples were obtained by brachial venipuncture and stored in Queen's lysis buffer (Seutin, White, & Boag, 1991).

### 2.2 Genomic hybrid index

We extracted genomic DNA from each sample either using a standard phenol‐chloroform extraction or with a Qiagen^®^ DNeasy kits (Qiagen, Valencia, CA) and determined the final concentration of each extraction using Qubit Fluorometric Calibration (QFC; Invitrogen, Carlsbad, CA). To estimate a high‐resolution genomic hybrid index, we used a double digest restriction association DNA sequencing (ddRAD) protocol following Peterson, Weber, Kay, Fisher, and Hoekstra (2012) with the modifications outlined in Campagna, Gronau, Silveira, Siepel, and Lovette (2015). We sequenced the two lanes of an Illumina HiSeq 2000 (150 bp, single‐end) at the Cornell University Life Sciences Core Laboratories Center (Ithaca, NY).

We demultiplexed sequencing reads within each index group using the barcode‐splitting program Sabre ( https://github.com/najoshi/sabre), allowing for one mismatch in the barcode plus enzyme cut‐site sequence. We used BOWTIE2 (Langmead & Salzberg, 2012) to map each of the individual reads to a build of the myrtle warbler genome (Toews, Brelsford, Grossen, Milá, & Irwin, 2016), using the “very sensitive local” set of alignment presets. For SNP discovery and variant calling, we used the UnifiedGenotyper in GATK (DePristo et al., 2011), and used GATK and VCFtools (Danecek et al., 2011) to apply the quality filters outlined in Toews, Taylor et al. (2016). We coded genotypes with a Phred‐scaled quality lower than 20 as missing data and excluded loci with more than 30% missing data and/or a minor allele frequency of less than 1%.

To estimate hybrid ancestry, we used the program STRUCTURE (version 2.3.4, Falush, Stephens, & Pritchard, 2003). For this analysis, we focused on markers that were spaced at least 10 kb apart using the “thin” function in VCFTools (Danecek et al., 2011). We then ran STRUCTURE on this subset of SNPs (*n* = 4,661 loci) with *K* = 2 for 100,000 MCMC steps following a burn‐in of 100,000 iterations.

### 2.3 Determination of haemosporidian parasites

Infections by haemosporidian parasites were diagnosed by performing nested PCR as described in Jenkins, Delhaye, and Christe (2015), modified from Hellgren, Waldenström, and Bensch (2004). Briefly, the first PCR round was conducted using HaemNF1 and HaemNR3 primer pair in order to amplify a 617 bp conserved region of the haemosporidian (*Plasmodium*,* Haemoproteus,* and *Leucocytozoon*) *cytochrome b* (*cytb*) gene. We then amplified a 479 bp region from 1 μl of the product of the first PCR round, using the *Plasmodium*‐ and *Haemoproteus*‐specific HaemF and HaemR2 primers pair and the *Leucocytozoon*‐specific HaemFL and HaemR2L primers pair. PCR products were then visualized after agarose gel electrophoresis (2%). Positive PCR products were sequenced in both directions by Sanger sequencing (Microsynth AG, Balgach, Switzerland).

Double‐peak(s) in a chromatogram meant that several haemosporidian lineages were present in the sample (Bensch et al., 2009); these mixed infections were excluded from diversity analyses. Sequences were blasted against the GenBank^®^ and MalAvi databases (Bensch et al., 2009). Sequences that did not match at 100% of identity with any deposited sequence were named SETCOR03 to SETCOR19.

### 2.4 Sequence analyses and phylogenetic reconstruction

In order to compute phylogenetic diversity metrics, we reconstructed a phylogeny of the sampled haemosporidian lineages. We collated forward and reverse sequences using MEGA7 (Kumar, Stecher, & Tamura, 2016; Tamura, Dudley, Nei, & Kumar, 2007) and aligned the consensus sequences with ClustalW (Thompson, Higgins, & Gibson, 1994). We determined the best model of nucleotide substitution with the function “phymltest” (Guindon & Gascuel, 2003; Posada & Crandall, 2001) implemented in the package “ape” (Paradis, Claude, & Strimmer, 2004) in R version 3.1.1 (R Core Team, 2014). Phylogenetic trees were constructed using PhyML version 3.0 (Guindon et al., 2010) under the GTR + Γ model of nucleotide substitution. The topology robustness was assessed with 1,000 bootstraps.

### 2.5 Parasitaemia measurements

We performed quantitative PCR on samples infected by hDENPEN02, the most prevalent haemosporidian lineage in our samples, to measure relative parasitaemia. In order to do so, we designed a pair of primers to amplify a 101 bp fragment of *Haemoproteus cytb* mitochondrial gene: DENPEN02_cytb_Fw (5′‐CCGCTTTTATGGGTTATGTATTAC‐3′) and DENPEN02_cytb_Rev (5′‐CCATGAAACAAGTCCAGGTATA‐5′) and a specific TaqMan probe DENPEN02_cytb_Pr (FAM‐cytb‐BHQ1: 5′‐AACGGTTGCACCCCAGAAACTCATTTG‐3′). To use as an internal control, we designed a pair of primers to amplify a 115 bp fragment of *S. c. coronata* and *auduboni* RPL30 nuclear gene: DcRPL30F (5′‐GTCTGCAAGTGCTGAATCCT‐3′) and DcRPL30R (5′‐TGTGGCTCAGGAACCTTTAC‐3′) and a specific TaqMan probe DrRPL30Pr (CY3‐RPL30‐BHQ2: 5′‐GAGCCTGAGTAGGAGCAGCCTGA‐3′). Host and parasite genes were amplified in the same reaction, in duplicates.

A series of twofold dilutions of three samples (starting from 10 ng/μl) was used to establish a standard curve, computed as the mean Ct as a function of the common logarithm of the concentration. For a qPCR of 100% efficiency, the slope of the standard curve is −3.32. We validated the qPCR protocol when we obtained slopes between −3.2 and −3.8 for both parasite and host genes.

Reactions were run in a final volume of 20 μl, including 10 μl of Takyon Low ROX Probe 2X MasterMix (Eurogentec, Seraing, Belgium), 4 μl of genomic DNA (5 ng/μl), 0.5 μM of each primer, 0.2 μM of each probe, and 1.2 μl of ultrapure water. qPCR was performed in a 7500 real‐time PCR System (Applied Biosystem, Foster City, CA, USA) with the following thermal profile: 2 min at 50°C, 15 min at 95°C, followed by 48 cycles of 15 s at 95°C, and 1 min at 54°C (annealing temperature). In each run of the qPCR, three heavily infected samples were used as positive controls for reproducibility, as well as the standard curve, in duplicates. Ct value was estimated as the mean of the two replicates.

Host and parasite DNA concentrations (*α*) were calculated as:

$$\alpha =10^{\frac{C_{t}-I}{m}}$$

*C*_t_ being the mean of the measured *C*_t_, *I* the intercept of the standard curve, and *m* the slope of the standard curve. Relative parasitaemia (*R*) was calculated as the ratio between parasite and host DNA:

$$R=\frac{\alpha_{\mathrm{parasite}}}{\alpha_{\mathrm{host}}}$$

*R* was log‐transformed in order to normalize the distribution.

### 2.6 Analysis of the probability and intensity of infection

We used generalized linear mixed models (GLMMs) to examine the influence of hybrid index, elevation (as a proxy for associated environmental variables), and scaled mass index (SMI, as a proxy for bird body condition; Peig & Green, 2009) on probability of infection and coinfection in the hybrid zone, using site and transect of sampling as random factors, with the function “glmer” implemented in the R package “lme4” (Bates, Mächler, Bolker, & Walker, 2015). We fitted different models for five different response variables (1) *Plasmodium* infections, (2) *Haemoproteus* infections, (3) infections by hDENPEN02 (the most prevalent *Haemoproteus* lineage in the dataset), (4) *Leucocytozoon* infections, and (5) coinfection by *Plasmodium* and/or *Haemoproteus* and *Leucocytozoon*. In models (1) and (2), individuals showing *Plasmodium* and/or *Haemoproteus* mixed infections were excluded as they were unidentifiable. We constructed a maximal model containing the presence/absence of infection (coinfection for model 5) as response variable with a binomial error structure, explanatory variables as linear (hybrid index, elevation and SMI) and quadratic (hybrid index and elevation) functions and the interaction between hybrid index and elevation (only linear) to account for differences in performance of each subspecies/hybrids at different elevations. We used the likelihood ratio test to drop nonsignificant terms, starting with the interaction, and then the quadratic terms, but in every case, we kept at least the three explanatory variables as linear functions in the final models. The reason for this is that Crawley (2012) suggests reporting values from the maximal model when the data are nonorthogonal, as is often the case in observational studies, but also generally recommends keeping the models simple by removing interactions and higher order terms if they are not significant.

We used linear mixed models to examine the variation in hDENPEN02 parasitaemia in the hybrid zone dependent on host hybrid index, elevation, scaled mass index, and using sites and transects of sampling as random factors (model 6), with the function “lmer” implemented in the R package “lme4” (Bates et al., 2015). We selected the best model according to the same procedure of the analyses of the infection probability.

As data used in models 1–6 were sampled along transects, we computed Moran's I (Gittleman & Kot, 1990) to test for spatial autocorrelation in the residuals of these models with the function “Moran.I” implemented in the R package “ape” (Paradis et al., 2004).

To assess the relative importance of age, sex, SMI, and hybrid index on infection probability in the migrating birds sampled in 2015, we fitted generalized linear models (GLMs) using these explanatory variables and the absence/presence of infection as a response variable with a binomial error structure, with the function “glm” implemented in the R package “lme4” (Bates et al., 2015). We tested the interactions between age and sex, as well as the hybrid index as a quadratic function, and selected the best model according to the same procedure as above. We fitted different models for (7) *Plasmodium* infections, (8) *Haemoproteus* infections, (9) *Leucocytozoon* infections, and (10) coinfection by *Plasmodium* and/or *Haemoproteus* and *Leucocytozoon*. In models (7) and (8), individuals showing *Plasmodium* and/or *Haemoproteus* mixed infections were excluded as they were unidentifiable.

### 2.7 Diversity analyses

Analyses regarding diversity of haemosporidian lineages were conducted on the whole sampling region and considering *Plasmodium* and *Haemoproteus* infections together, because the number of *Plasmodium* infections was low, and because *Plasmodium* and *Haemoproteus* groups cluster together in the phylogeny; *Leucocytozoon* infections were analyzed separately. We tested our hypothesis that genetically similar birds had more similar parasite lineages. In order to do this, we conducted a Mantel test using the “mantel.rtest” function implemented in the R package “ade4” (Dray & Dufour, 2015) with 10,000 permutations. We also did a partial Mantel test, correcting for geographic distances, using the “mantel.partial” function implemented in the R package “vegan” (Oksanen et al., 2015) with the Pearson's method.

To compare the diversity of haemosporidian lineages between subspecies of yellow‐rumped warblers and the hybrids, we calculated the Simpson's diversity index (D; Simpson, 1949) of lineages in each category of host (*S. c. coronata*,* S. c. auduboni*, hybrids), computed as $\sum {\left(\frac{n_{i}}{N}\right)}^{2}$ (*n*_*i*_ being the number of individuals of lineage *i* and *N* the total number of individuals) in the R package “vegan” (Oksanen et al., 2015). We report 1‐D so higher values represent a higher diversity. To account for the effect of phylogeny, we also calculated the standardized effect size (SES) of the Mean Pairwise Phylogenetic Distance (MPD) and the SES of the Mean Nearest Taxon Distance (MNTD; Webb, Ackerly, Mcpeek, Donoghue, & Webb, 2002) as metrics of phylogenetic diversity, with the functions “ses.mpd” and “ses.mntd” implemented in the R package “picante” (Kembel et al., 2010). We used 10,000 permutations to create the null models. SES_MPD_ and SES_MNTD_ are equivalent to −1 times the Net Relatedness Index (NRI) and −1 times the Nearest Taxon Index (NTI), respectively. NRI detects phylogenetic clustering or evenness patterns across the whole tree, while NTI is more sensitive toward the tips of the tree. Clustering means that phylogenetically closely related lineages are found more often in the same host species than expected by chance. Evenness means that the co‐occurring lineages are distributed more evenly in the tree than expected by chance. We calculated these metrics using the subspecies of warbler as “communities” in order to determine whether hybrids have a higher diversity of parasites than the parental subspecies, in which case we would expect negative values of NRI and NTI in hybrids (i.e., they contain lineages scattered across the tree), and positive values in parental subspecies if they are infected by specific lineages.

## 3 RESULTS

### 3.1 Analysis of the probability and intensity of infection

Among the 617 yellow‐rumped warblers screened for haemosporidian parasites, 345 birds were sampled from the hybrid zone. In the hybrid zone, we observed a prevalence of 3.2% of *Plasmodium* infections (*n* = 11), 30.1% of *Haemoproteus* infections (104), and 2.3% (8) of *Plasmodium* and/or *Haemoproteus* mixed infections. There was 45.8% of *Leucocytozoon* infections (*n* = 158) and 9.9% of *Leucocytozoon* mixed infections (34) and 23.2% of birds (80) were coinfected by parasites of both *Leucocytozoon* and *Plasmodium* and/or *Haemoproteus* genera. Nine *Plasmodium* and/or *Haemoproteus* and 10 *Leucocytozoon* infections could not be identified due to poor‐quality sequences and were therefore excluded from further analysis.

**Figure 3:**
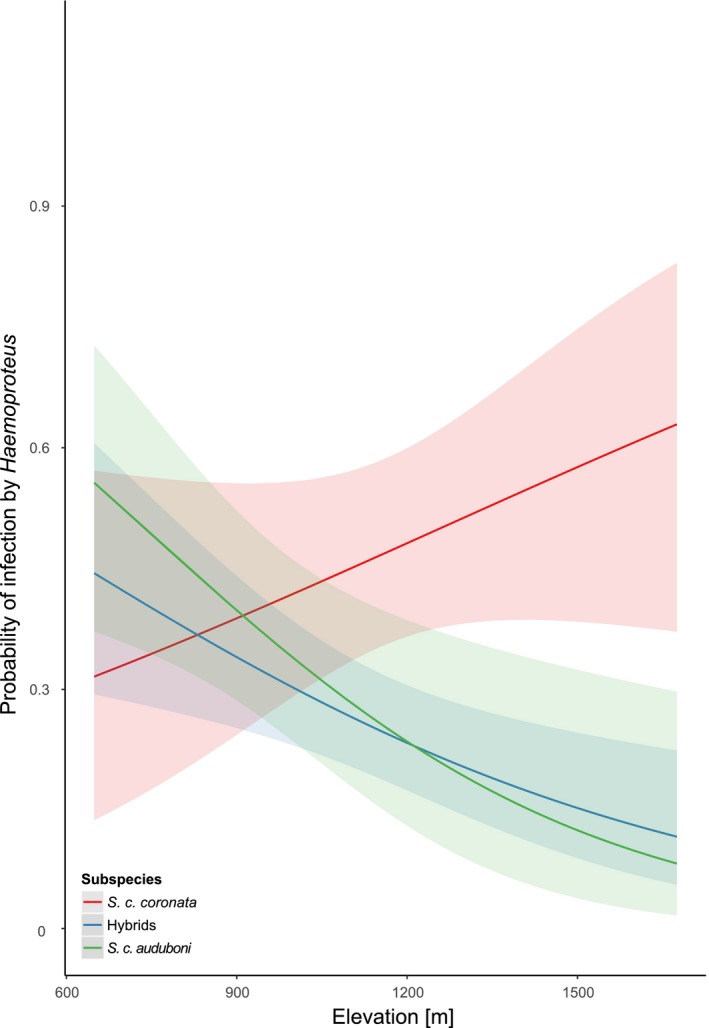
Probability of infection by *Haemoproteus* in relation to elevation (in m) for each yellow‐rumped warbler group. For the sake of clarity, we plot values predicted by a model that uses hybrid index of yellow‐rumped warblers as categories instead of the continuous variable “hybrid index” presented in the results section. Except for this difference, the model has the same structure to model (2) in Table 1

The results of the models to test elevation and hybrid index on probability of infection in the hybrid zone are reported in Table 1. We found that the probability of infection by *Haemoproteus* varies according to an interaction between elevation and hybrid index (model 2: $\chi_{1}^{2}$ = 6.36, *p *=* *0.012): The probability of infection decreases with elevation in *S. c. auduboni* and hybrids, *S. c. coronata* are more likely to be infected at higher elevation (Figure 3). This effect seems mainly driven by hDENPEN02 (model 3: $\chi_{1}^{2}$ = 5.23, *p* = 0.022), a *Haemoproteus* lineage that was responsible for 96.1% (*n* = 100) of single *Haemoproteus* infections in the hybrid zone. Body condition also correlates the probability of infection by *Haemoproteus* (model 2: $\chi_{1}^{2}$ = 4.98, *p* = 0.026; model 3: $\chi_{1}^{2}$ = 5.93, *p* = 0.015 when considering only hDENPEN02 infections), probability of coinfection by *Leucocytozoon* and *Plasmodium* and/or *Haemoproteus* (model 5: $\chi_{1}^{2}$ = 9.02, *p *=* *0.003) and, marginally, by the probability of infection by *Plasmodium* (model 1: $\chi_{1}^{2}$ = 3.78, *p *=* *0.052): Infected birds were generally heavier than noninfected ones. In terms of elevation, only the quadratic function of elevation is associated to the probability of infection by *Leucocytozoon* (model 4: $\chi_{1}^{2}$ = 4.61, *p* = 0.032, Supporting Information Figure S1).

**Table 1:** Description of fitted models

Response variable	Explanatory variables	Deviance	AIC	$\chi_{1}^{2}$	Pr($\chi_{1}^{2}$)	Estimate	*SE*
(1) *Plasmodium* infections	Intercept					−15.53	6.72
	Elevation:hybrid index		106.67	1.93	0.165		
	I(elevation^2^)		105.11	0.44	0.509		
	I(hybrid index^2^)		105.14	0.47	0.494		
	**Elevation**		101.73	0.15	0.701	0.56	1.15
	**Hybrid index**		102.05	0.47	0.491	−0.38	0.55
	**SMI**		105.36	3.78	0.052	0.97	0.49
(2) *Haemoproteus* infections	Intercept					−9.12	3.99
	**Elevation:hybrid index**		366.34	6.36	0.012*	−2.35	0.96
	I(elevation^2^)		361.53	3.13	0.077		
	I(hybrid index^2^)		360.88	2.48	0.115		
	**Elevation**		Marginal			1.45	1.65
	**Hybrid index**					2.25	1.08
	**SMI**		364.96	4.98	0.026*	0.58	0.26
(3) Infection by DENPEN02	Intercept					−9.84	4.01
	**Elevation:hybrid index**		358.56	5.23	0.022*	−2.1	0.94
	I(elevation^2^)		354.41	3.65	0.056		
	I(hybrid index^2^)		353.79	3.02	0.082		
	**Elevation**		Marginal			1.54	1.67
	**Hybrid index**					1.88	1.06
	**SMI**		359.26	5.93	0.015*	0.64	0.26
(4) *Leucocytozoon* infections	Intercept					3.5	3.88
	Elevation:hybrid index		454.72	1.02	0.312		
	**I(elevation** ^**2**^ **)**		457.33	4.61	0.032*	5.03	2.09
	**I(hybrid index** ^**2**^ **)**		452.84	0.12	0.726	0.13	0.36
	**Elevation**		458.47	5.74	0.017*	−12.89	4.76
	**Hybrid index**		452.72	0	0.992	0.01	0.77
	**SMI**		455.07	2.35	0.126	0.32	0.21
(5) Coinfection by *Leucocytozoon* and *Plasmodium* and/or *Haemoproteus*	Intercept					−8.42	3.5
	Elevation:hybrid index		348.53	1.1	0.295		
	I(elevation^2^)		346.56	0.03	0.855		
	I(hybrid index^2^)		350.16	3.63	0.057		
	**Elevation**		349.39	3.16	0.076	−1.81	0.83
	**Hybrid index**		346.32	0.09	0.765	−0.08	0.27
	**SMI**		355.25	9.02	0.003**	0.76	0.26
(6) DENPEN02 parasitaemia	Intercept					3.7	3.02
	Elevation:hybrid index		99.86	2.29	0.131		
	**I(elevation** ^**2**^ **)**		101.92	4.05	0.044*	3.3	1.78
	**I(hybrid index** ^**2**^ **)**		99.58	1.72	0.190	−0.38	0.33
	**Elevation**		101.33	3.46	0.063	−6.7	3.94
	**Hybrid index**		99.12	1.25	0.263	0.62	0.67
(7) *Plasmodium* infections	Intercept					−6.6	12.1
	I(hybrid index^2^)	28.444	38.444	2.76	0.097	−1.48	1.26
	**Hybrid index**	29.546	37.546	1.1	0.294		
	Sex:age	25.688	37.688	3.04	0.081	M: −0.79	1.24
	**Sex**	28.839	36.839	0.39	0.530	HY: −2.01	1.29
	**Age**	31.405	39.405	2.96	0.085		
	**SMI**	28.67	36.67	0.23	0.635	0.53	1.05
(8) *Haemoproteus* infections	Intercept					−4.15	6.31
	I(hybrid index^2^)	83.617	93.617	0.45	0.501	−1.57	0.67
	**Hybrid index**	90.324	98.324	6.71	0.010**		
	Sex:age	83.163	95.163	1.4	0.237	M: −0.50	0.68
	**Sex**	84.167	92.167	0.55	0.458	HY: 0.21	0.7
	**Age**	83.668	91.668	0.05	0.821		
	**SMI**	84.103	92.103	0.49	0.486	0.35	0.55
(9) *Leucocytozoon* infections (single or mixed)	Intercept					−3.91	4.99
	I(hybrid index^2^)	125.46	135.46	1.1	0.295	0.91	0.6
	**Hybrid index**	128.15	136.15	2.69	0.101		
	Sex:age	124.36	136.36	0.11	0.741	M: −0.66	0.51
	**Sex**	127.06	135.06	1.6	0.205	HY: −0.14	0.5
	**Age**	125.5	133.5	0.04	0.841		
	**SMI**	125.69	133.69	0.24	0.628	0.22	0.43
(10) Coinfection by *Leucocytozoon* and *Plasmodium* and/or *Haemoproteus*	Intercept					4.67	8.32
	I(hybrid index^2^)	55.851	65.851	0.04	0.844	0.59	1.01
	**Hybrid index**	56.187	64.187	0.34	0.562		
	Sex:age	55.812	67.812	3.63	0.057	M: 0.50	0.94
	**Sex**	56.143	64.143	0.29	0.589	HY: −0.72	0.81
	**Age**	56.546	64.546	0.69	0.405		
	**SMI**	56.667	64.667	0.82	0.366	−0.66	0.73

*Notes*

Generalized linear mixed models (1)–(5) and generalized linear models (7)–(10) were fitted with a binomial error structure (logit link function). Response variable values were 1 if infected and 0 if uninfected in (1)–(4) and (7)–(9), 1 if coinfected and 0 if single or uninfected in (5) and (10), and the logarithm of parasitaemia in (6). Models (1)–(6) had the transect and site of sampling as random factors. We performed likelihood ratio tests to remove nonsignificant variables in the following order: (a) interaction; and (b) quadratic terms, both kept if at least one of them was significant. If not significant, we report values obtained after removal of this term from the last model that contained it. Explanatory variables in bold are those that were kept in the final model. In every case, elevation, hybrid index and scaled mass index (SMI) were kept in the final model as our data are nonorthogonal, as suggested by Crawley (2012).

*p‐value <0.05; **p‐value <0.01.

**Figure 4:**
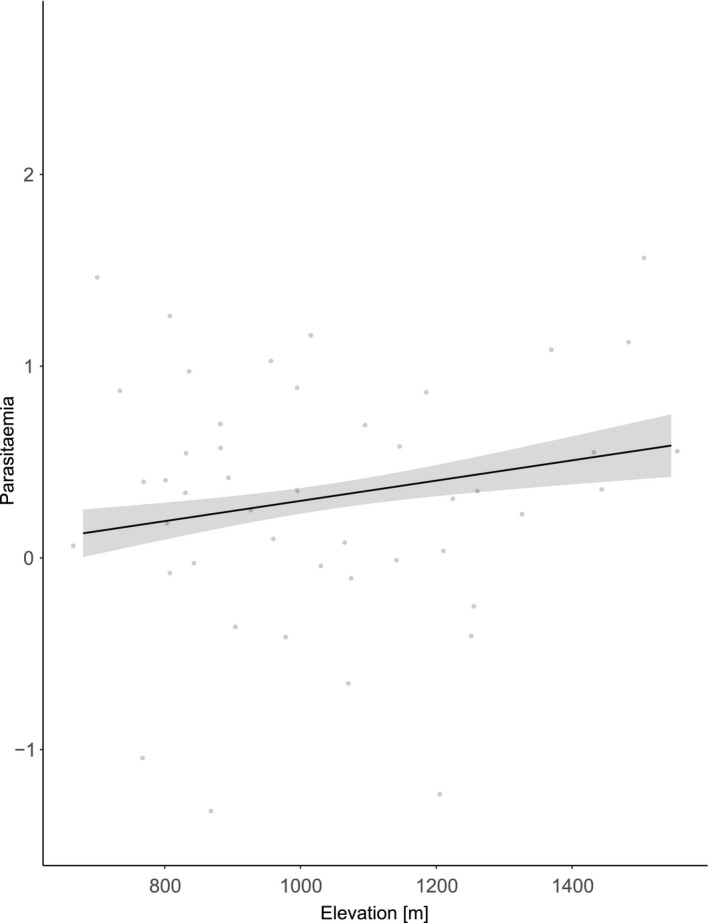
Parasitaemia in birds infected by hDENPEN02 as a function of elevation (m)

A quadratic function of elevation also best predicted parasitaemia of birds infected by hDENPEN02 (model 6: $\chi_{1}^{2}$ = 4.05, *p *=* *0.044), and hybridization status did not influence it, nor the body condition (Table 1 and Figure 4). We found no spatial autocorrelation in the residuals of the models (Supporting Information Table S2).

**Figure 5:**
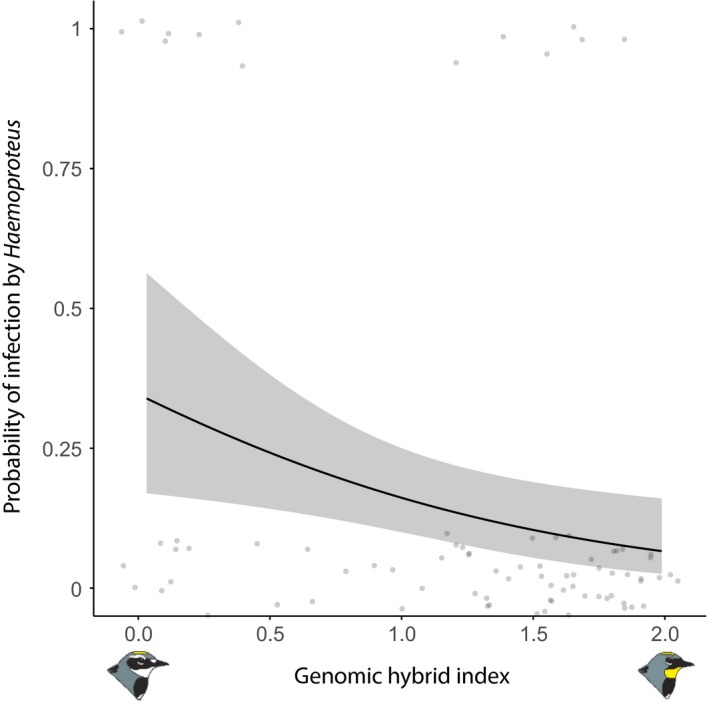
Probability of infection by *Haemoproteus* as a function of genomic hybrid index in migrating birds sampled in 2015. Hybrid index: 0 represents pure *Setophaga coronata coronata*, 2 represents pure *Setophaga coronata auduboni*; values between 0 and 2 are admixed individuals. Bird drawings from Milá, Toews, Smith, and Wayne (2011)

In birds sampled in autumn 2015 during autumn migration, neither age, sex, nor body condition influenced the probability of infection. However, genomic hybrid index was significantly associated with the probability of infection by *Haemoproteus*:* S. c. coronata* was more infected than *S. c. auduboni* and hybrids (model 8: $\chi_{1}^{2}$ = 5.52, *p *=* *0.010; Table 1, Figure 5).

### 3.2 Diversity analysis

**Figure 6:**
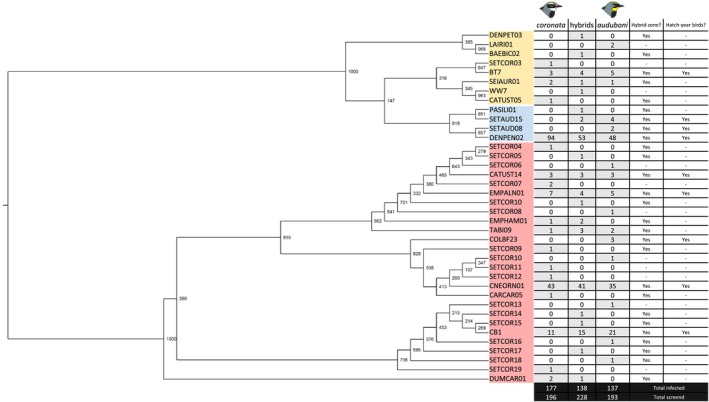
Phylogenetic tree of lineages found in the sampling region. Ultrametric phylogenetic tree of haemosporidian lineages found in yellow‐rumped warblers in a given sampling region, and the number of birds infected by each lineage. Node labels represent bootstrap values (1,000 replicates). Yellow shading: *Plasmodium* lineages; blue shading: *Haemoproteus* lineages; red shading: *Leucocytozoon* lineages. Last columns indicate whether the lineage was found in the hybrid zone and whether the lineage was found in hatch‐year birds migrating for the first time in 2015, and thus that are transmitted on the breeding ground. Bird drawings from Milá et al. (2011)

Eight different lineages of *Plasmodium*, four different lineages of *Haemoproteus*, and 26 different lineages of *Leucocytozoon* were found in infected birds in the whole sampling region (Figure 6). The lineages responsible for mixed infections could not be identified. Three lineages in particular were abundant: Among identified *Haemoproteus* infections, 95.6% were hDENPEN02 and among identified *Leucocytozoon* infections, 52.9% were lCNEORN01, and 20.9% were lCB1, which match morphospecies *Leucocytozoon majoris*. We found 17 haplotypes that did not match at 100% of identity the sequences referenced in GenBank nor in MalAvi database of avian malaria lineages (Bensch et al., 2009). In total, eight lineages of the haemosporidian parasites that we found are shared between the two subspecies and the hybrids. One *Plasmodium* lineage, three *Haemoproteus* lineages, and five *Leucocytozoon* lineages were found in migrating hatch‐year birds in 2015; all these lineages had been found in the birds from the first sampling (Figure 6).

There was no correlation between host hybrid index and lineage composition (*Plasmodium*/*Haemoproteus*:* R *=* *0.02, *p *=* *0.16; *Leucocytozoon*:* R *=* *0.01, *p *=* *0.26), including when correcting for the effect of geographic distance (Partial Mantel test, *Plasmodium*/*Haemoproteus*:* R *=* *0.02, *p *=* *0.17; *Leucocytozoon*:* R *=* *0.00, *p *=* *0.47).

Measures of Simpson's diversity did not show a higher diversity of haemosporidian lineages in hybrids (Table 2). Similarly, the values of NRI and NTI for *Plasmodium* lineages in hybrids were negative and smaller than in pure individuals (Table 2), suggesting a tendency for phylogenetic evenness, a higher diversity than expected by chance, although note that these values were not significant. Only the NRI of *Plasmodium*/*Haemoproteus* lineages in *S. c. coronata* and the NTI of *Leucocytozoon* lineages in *S. c. auduboni* were significantly positive, suggesting basal phylogenetic clustering of *Plasmodium* lineages in *S. c. coronata* and a terminal phylogenetic clustering of *Leucocytozoon* lineages in *S. c. auduboni*. In other words, *S. c. coronata* tend to be infected by *Plasmodium* lineages that are phylogenetically closer than expected by chance and the same with *Leucocytozoon* lineages in *S. c. auduboni*.

**Table 2:** Diversity analyses results

	1‐D	NRI	NTI
*Plasmodium*/*Haemoproteus*			
*coronata*	0.13	**1.78**	−0.39
Hybrids	0.31	−1.58	−1.27
*auduboni*	0.39	−0.31	0.88
*Leucocytozoon*			
*coronata*	0.65	1.62	−1.10
Hybrids	0.64	−0.46	0.66
*auduboni*	0.69	−0.97	**1.64**

*Note*

Simpson's index of diversity (D) presented as 1‐D (so higher values reflect higher diversity), Net Relatedness Index (NRI) and Nearest Taxon Index (NTI) of haemosporidian parasites in hosts subspecies and their hybrids. Values in bold represent significant values (*p *<* *0.05).

## 4 DISCUSSION

In this study, we broadly sampled a naturally occurring hybrid zone between two yellow‐rumped warbler subspecies to explore the role of parasites in potentially selecting against hybrids. We found that haemosporidian parasites—in diversity and prevalence—are unlikely to play a major role in selecting against *S. c. coronata* × *S. c. auduboni* hybrids. Indeed, hybrids did not seem to be more infected by haemosporidian parasites than parental subspecies. We found that prevalence, coinfection probability, parasitaemia and diversity of haemosporidian lineages were not higher in hybrids, as we had originally predicted. In migrating yellow‐rumped warblers sampled in autumn, there was no effect of hybrid index on the probability of infection by *Plasmodium* or *Leucocytozoon*, but we found that *S. c. coronata* had a higher probability to be infected by *Haemoproteus* than *S. c. auduboni* and hybrids.

From our results, it seems that most haemosporidian lineages were shared between myrtle and Audubon's warblers. Indeed, three lineages were very abundant in *S. c. coronata*,* S. c. auduboni*, and their hybrids. Some other lineages were found only in one subspecies, or only in hybrids, or in one subspecies and in hybrids, but this was generally restricted to only one or two individuals. Additional sampling would be required to determine whether these lineages, when undocumented, are rare and specific, or if they are simply rare and exclusivity is due to stochastic sampling effects. According to theory on the evolution of specialization, we could expect specialist haemosporidian parasites to show a higher prevalence and virulence than generalists (Futuyma & Moreno, 1988). This is sometimes the case for virulence: For example, Garamszegi (2006) showed that specialist malaria parasites of primates had a higher parasitaemia (used as a proxy for virulence) than generalists. However, Hellgren, Pérez‐Tris, and Bensch (2009) showed that overall more generalist *Plasmodium* and *Haemoproteus* parasites also were the most abundant in single subspecies. In addition, the haemosporidian lineages found in the sampled yellow‐rumped warblers are relatively generalist and have been found in other species in the MalAvi database (Bensch et al., 2009). For example, hDENPEN02 has been found in at least seven other species of Passeriformes in North America and lCNEORN01 in ten (Oakgrove et al., 2014; Outlaw & Ricklefs, 2009; Ricklefs & Fallon, 2002; Walther et al., 2016). Given these results, we suggest that it is unlikely that these parasites specialize and thereby exert differential levels of selection between myrtle and Audubon's warblers. In addition, the common lineages we found in the hybrid zone were present in both after hatch‐year and hatch‐year birds, which had not completed a full annual migration cycle yet. This means these young birds were infected on the breeding ground, as opposed to their wintering grounds, suggesting that infection occurs in the nest or soon after fledging. This is supported by a recent study on *Setophaga coronata auduboni* in New Mexico (US) sky islands that found a high diversity of haemosporidian lineages but no bird infected by hDENPEN02 (Williamson et al., 2018).

In sampling of yellow‐rumped warblers along five transects across the Rocky Mountains, we documented a consistent pattern: Elevation was an important predictor of prevalence, especially with respect to *Leucocytozoon* infections. Indeed, the probability of infection by *Leucocytozoon* decreased with elevation in pure individuals and hybrids. In the context of studying factors shaping haemosporidian parasites distribution in general, especially regarding the importance of predicting biodiversity and shifts in community structures under climatic change, it would be of major value to determine whether this pattern is a result of an effect of elevation, geographic position and their correlated abiotic factors (e.g., temperature, solar radiation, humidity, snow cover) or an effect of correlated biotic factors (e.g., change in plant and animal communities). Future studies on elevational gradients of parasite distribution should test whether these effects specifically act on bird susceptibility, parasite distribution, or on vector distribution and preferences. We propose that the presence of suitable conditions for vectors was the main driver for the observed *Leucocytozoon* distribution. Black flies (Simuliidae), the vectors of *Leucocytozoon* parasites, depend on running water bodies for their reproduction, and so streams and rivers are critical to their distribution (Crosskey, 1990). Temperature is also a factor that influences larval survival (Ross & Merritt, 1978). Many studies focusing on other regions found strong elevation‐dependent structuring of *Leucocytozoon* (González et al., 2014; Imura et al., 2012; van Rooyen et al., 2013; Zamora‐Vilchis et al., 2012).

Regarding the pattern of *Haemoproteus* prevalence, elevation alone is not a sufficient explanation, as it seemed to also interact with hybrid index. In particular, while there was a trend for decreasing prevalence in *S. c. auduboni* and hybrids, the probability of infection by *Haemoproteus* in *S. c. coronata* strongly increased with elevation. This is consistent with Williamson et al. (2018) who found that probability of infection by *Parahaemoproteus* decreased with elevation in New Mexico sky‐island Audubon's warblers. One possibility could be that *S. c. coronata* do not cope as well in high elevation environments. Stress induced by a suboptimal ability to acquire resources, for example, might enhance *S. c. coronata* susceptibility to *Haemoproteus* infection. This mechanism has been supported by the effect of food supplementation on *Plasmodium* parasitaemia in experimentally infected canaries (Cornet, Bichet, Larcombe, Faivre, & Sorci, 2014) and this effect might well be enhanced in the wild in interaction with other sources of stress.

This study also revealed a pattern, previously identified in great tits (Ots & Horak, 1998 but see Bennett, Caines, & Bishop, 1988), that birds infected by *Haemoproteus* have a higher body condition than uninfected birds. Indeed, we expected infected birds to have a lower body condition if haemosporidian infections were detrimental for their health. However, this pattern was observed only in the breeding birds sampled in spring, while in birds sampled in autumn 2015 at the beginning of their migration, there was no effect of the body condition on the probability of infection. It is possible that haemosporidian infections are more detrimental to yellow‐rumped warblers of poor condition, and that only individuals of good condition are able to survive the winter with an infection. We expect the weakest birds to die especially during the winter because migration is potentially stressful and requires substantial energy reserves (Wikelski et al., 2003). In addition, birds might be exposed to other parasite species on their wintering ground, which would be another source of physiological stress. Evidence of the impact of haemosporidian infections on yellow‐rumped warbler health and mortality would be useful to assess the validity of this hypothesis. It would also help us to conclude on the potential role of haemosporidian parasites in selection against *S. c. coronata* and *S. c. auduboni* hybrids, as even if hybrids were not more infected, they could suffer higher costs of being infected than their parental subspecies.

In conclusion, we found that haemosporidian parasites seem unlikely to play a major role in imposing a stronger selection pressure in hybrids of yellow‐rumped warblers. *S. c. coronata* × *S. c. auduboni* hybrids seem to exhibit similar patterns to *S. c. auduboni* with regard to how elevation affects their infection probability. We also found that both subspecies and hybrids share most of their haemosporidian lineages, which is consistent with these lineages being generalists. Finally, it seems that elevation, or other correlated environmental factors, has an important influence on haemosporidian prevalence, especially in *Leucocytozoon*. This study sheds some light on the role of haemosporidian parasites on speciation and opens the door for further investigations about the importance of parasites in driving host species diversification. Further work should investigate the potential effect of haemosporidian parasites on the fitness of both yellow‐rumped warblers and their hybrids.

## CONFLICT OF INTEREST

None declared.

## AUTHOR CONTRIBUTIONS

P.C., A.B., and T.J. conceived the idea. A.B., C.S.C., T.J., and D.P.L.T. collected the data. A.B., C.S.C., and D.P.L.T. performed the laboratory work. A.B., C.S.C., T.J., and D.P.L.T. performed the analysis. All authors discussed the results and contributed to the final manuscript.

## DATA ACCESSIBILITY

Upon acceptance of this manuscript, all data supporting this study will be made available on Dryad. All sequences will be made available on GenBank and MalAvi.
