# Supplementary material for: Prevalence and diversity of haemosporidian parasites in the yellow‐rumped warbler hybrid zone
Source: Ecol Evol. 2018 Sep 12;8(19):9834–47. doi: 10.1002/ece3.4469 (PMC6202724; doi:10.1002/ece3.4469)
Supplement: Supplementary file 1 [file ECE3-8-9834-s001.docx]

# Prevalence and diversity of haemosporidian parasites in the yellow-rumped warbler hybrid zone

Camille-Sophie Cozzarolo, Tania Jenkins, David P. L. Toews, Alan Brelsford* and Philippe Christe*

# Supplementary material


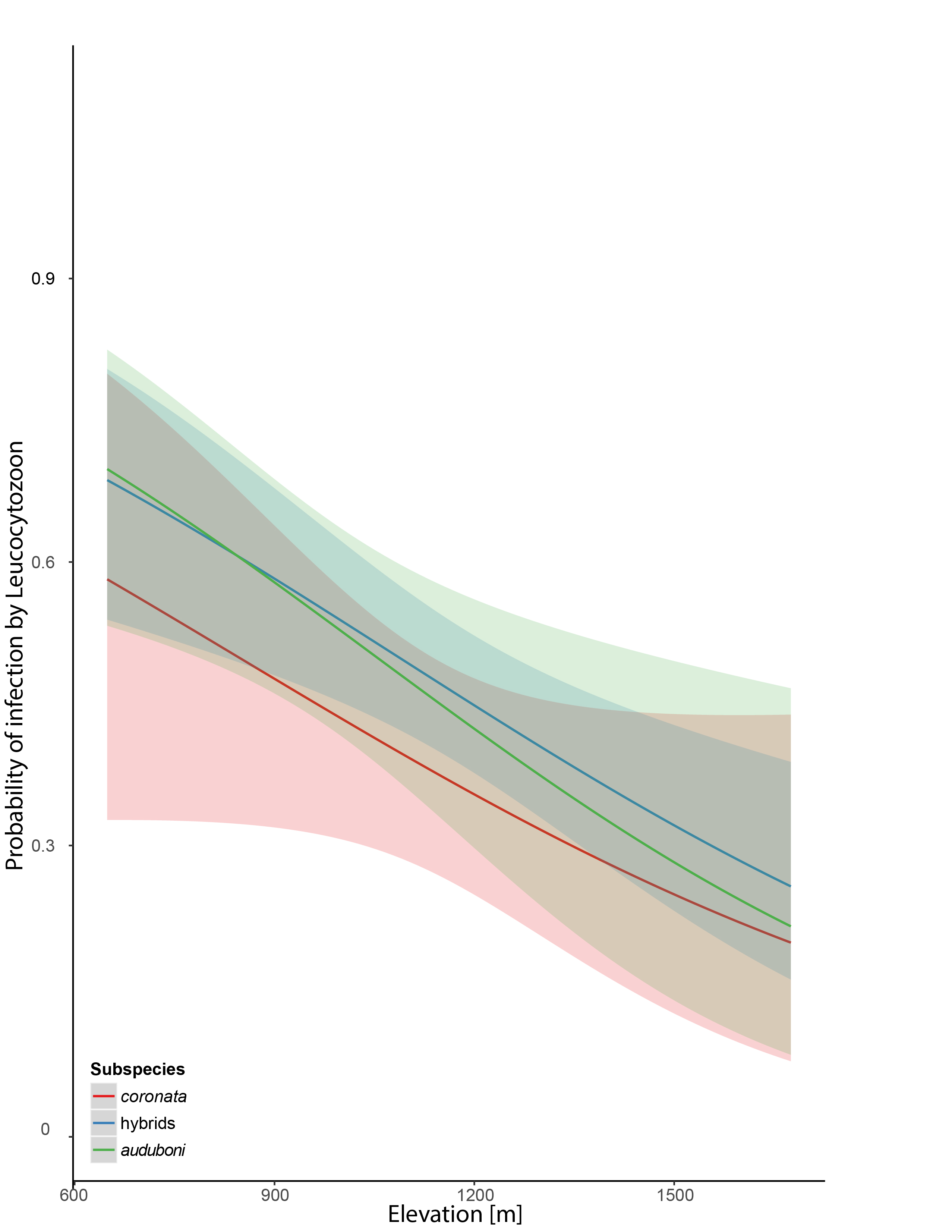


Figure S1 **Probability of infection by *Leucocytozoon* in relation to elevation (in m)** **for each yellow-rumped warbler group**. For the sake of clarity, we plot values predicted by a model that uses hybrid status of yellow-rumped warblers as categories instead of the continuous variable “hybrid index” presented in the results section. Except for this difference, the model has the same structure to model 4) in Table 1.

Table S1 Traits scored for the determination of the hybrid index (Hubbard 1969).

| Traits | *coronata* (0) | *auduboni* (2) |
| --- | --- | --- |
| throat colour | white | yellow |
| auricular colour | black | gray |
| white supraloral spot | present | absent |
| white postocular line | present | absent |
| wing pattern | two distinct white bars | single broad white patch |

Table S2 Moran’s I in models 1 to 6 (data sampled along transects)

|  | Moran’s I | p-value |
| --- | --- | --- |
| model 1 | -0.01954 | 0.511243 |
| model 2 | -0.03648 | 0.200137 |
| model 3 | -0.02929 | 0.314737 |
| model 4 | 0.011907 | 0.548921 |
| model 5 | -0.0209 | 0.465541 |
| model 6 | -0.04476 | 0.825582 |
